# Selective Metaphor Impairments After Left, Not Right, Hemisphere Injury

**DOI:** 10.3389/fpsyg.2018.02308

**Published:** 2018-12-03

**Authors:** Eileen R. Cardillo, Marguerite McQuire, Anjan Chatterjee

**Affiliations:** Department of Neurology and Center for Cognitive Neuroscience, University of Pennsylvania, Philadelphia, PA, United States

**Keywords:** metaphor, aphasia, focal lesion patients, figurative language, case study, sentence comprehension

## Abstract

The relative contributions of the left and right hemispheres to the processing of metaphoric language remains unresolved. Neuropsychological studies of brain-injured patients have motivated the hypothesis that the right hemisphere plays a critical role in understanding metaphors. However, the data are inconsistent and the hypothesis is not well-supported by neuroimaging research. To address this ambiguity about the right hemisphere’s role, we administered a metaphor sentence comprehension task to 20 left-hemisphere injured patients, 20 right hemisphere injured patients, and 20 healthy controls. Stimuli consisted of metaphors of three different types: predicate metaphors based on action verbs, nominal metaphors based on event nouns, and nominal metaphors based on entity nouns. For each metaphor (*n* = 60), a closely matched literal sentence with the same source term was also generated. Each sentence was followed by four adjective–noun answer choices (target + three foil types) and participants were instructed to select the phrase that best matched the meaning of the sentence. As a group, both left and right hemisphere patients performed worse on metaphoric than literal sentences, and the degree of this difficulty varied for the different types of metaphor – but there was no difference between the two patient groups. Tests for literal-metaphor dissociations at the level of single cases revealed two types of impairments: general comprehension deficits affecting metaphors and literal sentences equally, and selective metaphor impairments that were specific to different types of metaphor. All cases with selective metaphor deficits had injury to the left hemisphere, and no known comprehension difficulties with literal language. Our results argue against the hypothesis of a specific or necessary contribution of the right hemisphere for understanding metaphoric language. Further, they reveal deficits in metaphoric language comprehension not captured by traditional language assessments, suggesting overlooked communication difficulties in left hemisphere patients.

## Introduction

Humans are a loquacious lot. The average speaker obliges their listener to keep up with 150–190 words per minute (Tauroza and Allison, 1990) and the average college-aged reader consumes 300 words per minute (Sereno and Rayner, 2003). While our species’ unique capacity for language is news to none, what many may not appreciate is that one out of every seven or eight of these rapidly digested words is metaphorical (Pragglejaz Group, 2007). Despite its poetic associations, metaphor is frequently enlisted to expand and enrich our ability to express complex thoughts and feelings. When introducing an unfamiliar concept, a metaphor comparing the new domain to a familiar one is an effective teaching device familiar to every educator and parent (e.g., *The thalamus is a relay station*). If attempting to describe an idea or state of affairs without a clearly discernible referent in the world – as is the case with abstract concepts, social dynamics, and internal emotional states – a metaphor helpfully illuminates by reference to a more accessible one (e.g., *a tepid romance*). In other instances, a literal expression may exist and suffice, but a metaphor may be preferred for its ability to sharpen meaning, rouse a listener’s attention, and encourage particular inferences (Compare the literal statement “*The president’s opinion has changed over time*” with the metaphorical spins “*The president’s opinion has evolved*” or “*The president’s opinion has flip-flopped*”). The seeming ubiquity of metaphor in thought (Lakoff and Johnson, 1980) and language (Pragglejaz Group, 2007) necessitates that any account of how the human brain evolved to so effortlessly produce and understand literal language also explain the talking ape’s figurative finesse.

Since the pioneering work of Broca, Wernicke, and Lictheim (Roth, 2014), the specialization of the left hemisphere for supporting language comprehension and production has been widely accepted. However, patient research in the late 1970s and 1980s indicated the “quiet” right hemisphere also contributes to our linguistic abilities, suggesting a unique capacity for processing figurative language. When asked to match a sentence expressing a conventional metaphor to a picture (e.g., *It was such colorful music*), Winner and Gardner (1977) observed that RH patients were less likely to select the appropriate metaphoric picture than LH patients, showing a bias for a literal interpretation instead. A few years later, Brownell et al. (1984, 1990) reported that when presented with a triad of words and asked to make a semantic similarity judgment, RH patients were less likely to choose a metaphorically related word than were LH patients. Subsequent studies with brain injured patients also reported differences between RH patients and healthy controls on metaphoric conditions (Tompkins, 1990; Mackenzie et al., 1999; Gagnon et al., 2003; Klepousniotou and Baum, 2005b), strengthening the hypothesis that the right hemisphere plays a specific and necessary role in our ability to understand metaphor.

This idea was bolstered by observations that RH patients had impairments affecting other forms of non-literal language (for review, see Johns et al., 2008), and fit well with the hypothesis that the right hemisphere specializes in coding semantics coarsely (Beeman and Chiarello, 1998). An intact left hemisphere, optimized for rapid, fine-grained semantic associations, would be insufficient to successfully relate the semantically distant source and target terms of a metaphor. In contrast, the right hemisphere’s coarse-grained coding or sustained activation of broad semantic fields, would be ideally suited for such a task. Alternatively, Giora (1997) argued that the right hemisphere’s critical role in comprehending metaphor may reflect its specialization for deriving low-salience meanings, rather than figurativeness, *per se*. Insofar as literal, not metaphoric, meanings are dominant associations for most words, a RH processing preference for metaphoricity and low-salience would largely produce similar deficits after injury.

In light of this evidence and reasoning, neuropsychological assessment and therapy for right-hemisphere injured patients routinely targets an anticipated difficulty with metaphoric language. For example, the Right Hemisphere Language Battery (RHLB; Bryan, 1989) attempts to assess a variety of potential language impairments specific to RH patients, including metaphor comprehension. Other researchers have designed structured interventions to improve metaphor comprehension following brain injury, specifically designing their therapies with the theoretical deficits of RH patients in mind (e.g., Lundgren et al., 2006, 2011). By contrast, aphasia assessments commonly administered to left hemisphere patients – e.g., the Western Aphasia Battery (WAB; Kertesz, 1982), Boston Diagnostic Aphasia examination (BDAE; Goodglass and Kaplan, 1983), the Boston Naming Test (BNT; Goodglass et al., 1983), and Porch Index of Communicative Ability (Porch, 1971) – do not include measures of figurative language competence and this level of competence is not a routine target of speech-language rehabilitation.

The first neuroimaging study of metaphor comprehension (Bottini et al., 1994) bolstered the “Right Hemisphere Hypothesis” of metaphor that neuropsychological studies had inspired. However, the accumulated evidence from PET and fMRI studies since then no longer line up neatly in favor of this account. Rather, the neuroimaging literature suggests that metaphor comprehension is a bilaterally mediated and *left*-hemisphere dominant process. Meta-analyses of metaphoric versus literal language corroborate this impression: Bohrn et al. (2012), Rapp et al. (2012), and Yang (2014) all found a bilateral but strongly left-lateralized fronto-temporal network of areas more engaged by metaphors than literal expressions. More problematic, fine-grained analyses in all three studies indicate that right hemisphere engagement is driven by metaphors that are novel/low-salience – but the patient literature has relied on metaphors that are very familiar and presumably high-salience.

The neuroimaging literature casts doubt on a privileged role for the RH in metaphor, but the lack of convergence between the patient and neuroimaging literatures could also relate to a number of differences between the two experimental approaches. Foremost, the two methods enable different inferences about the neural areas they implicate. While neuroimaging studies can reveal areas engaged by a task requiring function X, they cannot tell us if those areas are necessary for that function. Studies of the same task in brain-injured patients can reveal necessary areas for function X, but are limited by the non-randomness of lesion locations (i.e., some areas are over- and under-represented) and the difficulty determining if an injured area is directly responsible for function X or its loss simply disrupts the connectivity between two (or more) areas that are necessary.

Methodological issues may also be at play. The patient literature on metaphor is, not surprisingly, more limited than the neuroimaging literature, and has been hampered at times by small numbers of items, tasks that introduce confounding variables, and limited specificity regarding patients’ lesions (Schmidt et al., 2010). The nature of metaphoric stimuli has also varied widely across both patient and neuroimaging studies, as have their attempts to balance their metaphorical and literal items for difficulty and other confounding differences (Cardillo et al., 2010, 2017). Notably, patient studies have frequently used word pairs or triplets to probe metaphor comprehension; whereas, imaging studies have predominantly used metaphors embedded within sentences. Patient studies also typically have older participants than imaging studies.

Lastly, the patient data point to more than one possible characterization of the RH’s possible special role in metaphor comprehension. The earliest studies (Winner and Gardner, 1977; Brownell et al., 1984, 1990) reported contrasting patterns of impairment between LH and RH patients, suggesting the RH plays a unique and critical role in appreciation of metaphoric meanings. However, although other patient studies replicated RH metaphor impairments, they did not replicate a dissociation by hemisphere, reporting instead comparable metaphor impairments in RH and LH patients (Tompkins, 1990; Gagnon et al., 2003). That neither hemisphere was sufficient for intact metaphor comprehension in these later studies suggests metaphor processing is bilaterally mediated rather than a special capacity of the RH, a hypothesis that aligns better with the neuroimaging literature.

The goal of the current study is to help resolve the outstanding ambiguity concerning the neural network necessary for metaphor comprehension. We attempt to reconcile the discrepant literature in several ways. First, we chose to leverage the powerful inferences enabled by patient research – i.e., that they can shed light on brain areas whose intact function is necessary for the cognitive dimension of interest rather than merely involved. Because metaphor is a complex cognitive process, and as such, likely relies on a distributed network of brain areas, we recruited a large group of patients (20 LH, 20 RH) irrespective of lesion location. In this way, we aimed to maximize our ability to detect critical areas of the metaphor-supporting neural network. Second, we used literal and metaphoric stimuli that have been extensively normed to avoid common confounds that can produce inadvertent difficulty differences between metaphoric and literal items. We also chose to test metaphor comprehension at the sentence level rather than using word pairs or triplets since this is more reflective of natural language and to bridge the gap between the stimuli used in patient versus neuroimaging studies.

Third, we chose a task that we have previously demonstrated to be optimized for studying metaphor in focal lesion patients (Ianni et al., 2014). Specifically, we used a metaphor multiple choice task that has the sensitivity to detect metaphor impairments in the absence of traditionally defined aphasia and the specificity to detect impairments of different types of metaphor. In this task, metaphors can be one of two different syntactic forms: nominal metaphors with noun source terms (*The X is a Y*), or predicate metaphors with verb source terms (*The A verb-ed the B*). Source terms can be from three possible semantic domains: entity nouns, event nouns, or action verbs. Each metaphor is matched to a literal sentence using the same source term. All sentences are followed by four possible two-word answer choices and patients are asked to select the answer that best matches the meaning of the sentence.

Our primary question concerns how injury to the right verses the left hemisphere impacts metaphoric and literal sentence comprehension. In the strongest formulation of the RH hypothesis, the RH plays a specific and critical role in metaphor comprehension and double dissociations between LH and RH patients are expected for literal and metaphoric comprehension. This account predicts that LH patients would exhibit impaired literal comprehension on our multiple choice task and RH patients would exhibit impaired metaphor comprehension. In a weaker version of the RH hypothesis, the RH plays a critical role in metaphoric comprehension, but in concert with the LH. This account predicts that RH patients will show greater difficulty with metaphors than literal sentences and that LH patients will be impaired on both, but not more so for metaphors than literal sentences.

A secondary question of interest is whether metaphors of different syntactic forms (nominal, predicate) or requiring abstractions from different semantic domains (entity nouns, event nouns, action verbs) differentially recruit the neural network for metaphor. We outlined elsewhere our reasons for suspecting that they might (see Cardillo et al., 2010, 2012, 2017; Jamrozik et al., 2016), but to our knowledge these distinctions have not been systematically considered within the same study. If metaphors of different types are understood using a common set of cognitive processes, then we predict patients with metaphor impairments will be equally impaired understanding metaphors of all three types. If different types of metaphor rely on different cognitive processes, then we anticipate metaphor impairments that selectively affect metaphors of a particular semantic domain or syntactic form but not others (e.g., nominals versus predicates or object semantics versus action/event semantics).

## Materials and Methods

### Subjects

**FIGURE 1:**
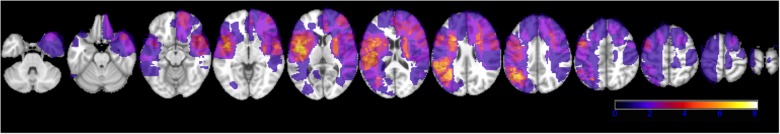
Overlay map depicting number of patients with damage in each voxel, presented in MNI space and following radiological convention (left side = right hemisphere).

Participants were 40 patients with chronic, unilateral focal lesions enrolled in the Center for Cognitive Neuroscience Focal Lesion Database (FOLD) at the University of Pennsylvania. Patients with a history of other neurological disorders, psychiatric disorders, or substance abuse are excluded from the database. For all patients, MRI or CT scans reviewed by a board-certified neurologist confirmed the presence of a focal lesion. Patients were selected irrespective of lesion location or behavioral deficits in order to sample brain areas of each hemisphere as completely as possible until a sample size of 20 patients with injuries in their left hemisphere (LH: Age = 60.2, *SD* = 11.9; Education = 14.3, *SD* = 2.3) and 20 patients with injuries in their right hemisphere (RH: Age = 62.8, *SD* = 11.4; Education = 14.4, *SD* = 2.6) was achieved. All participants were native English speakers, right-handed, and gave informed consent to participate in accordance with the Institutional Review Board of the University of Pennsylvania. LH and RH patients did not differ significantly in terms of age, education, lesion volume, or chronicity. Detailed demographic and neuropsychological information about the patients is provided in Tables 1, 2 and the distribution of lesions in standard space is provided in Figure 1.

**Table 1:** Demographic and neuropsychological profiles of LH cases.

ID#	Sex	Age	Education (years)	Lesion Side	Region	Lesion volume^1^	Etiology	Chronicity (months)	PBAC					MMSE^2^	AM-NART	WAB (AQ)^3^	OANB	
													
									Mem	VisSp	Lang	Exec	Beh				Axn	Obj
									(0–27)	(0–18)	(0–12)	(0–26)	(0–24)	(0–30)				
085	F	66	15	L	Ins	13079	Stroke	225	18	18	11	19.5	24	29	122.0	98.8	100.0	98.0
107	M	72	16	L	FP	33181	Stroke	192	–	–	–	–	–	–	103.0	-	-	-
141	F	53	16	L	Ins	21605	Stroke	154	–	–	–	–	–	–	113.0	98.8	96.0	100.0
215	M	64	14	L	F	17422	Stroke	174	18	17	11	18.5	24	29	106.0	94.4	93.8	96.0
236	M	66	18	L	FP	155982	Stroke	221	17.5	17	8.5	9.5	24	29	100.0	90.8	94.0	88.0
244	M	60	13	L	T/Cer	47240	Stroke	168	–	–	12	18.5	24	27	109.0	98.4	98.8	100.0
318	F	62	12	L	BG	20650	Stroke	160	19	18	12	19	24	29	112.0	99.0	100.0	96.0
360	M	60	12	L	T/BG	38063	Stroke	146	–	–	–	–	–	–	-	65.3	28.0	52.0
363	M	75	16	L	F	16845	Stroke	138	14	18	9	15.5	24	25	115.0	91.4	95.0	96.0
384	M	74	12	L	F	11306	HEM	143	14	13	10	19.5	24	22	110.0	91.3	98.8	100.0
428	M	58	12	L	F/CC	3592	Stroke	141	15.5	12	10.5	17.5	24	30	113.0	95.5	100.0	100.0
493	M	70	14	L	F	22404	ANR	122	10	18	10.5	15.5	24	24.5	104.0	92.1	95.0	98.0
529	F	68	12	L	F	8969	ANR	127	13	13	8	17.5	23	26	95.0	94.9	90.1	94.0
565	F	55	12	L	F	14517	ANR	125	23	17	11	23	24	30	104.0	99.8	97.5	98.0
642	M	78	12	L	TP	7996	Stroke	130	16	18	11	19	24	25	-	96.8	98.0	94.0
775	M	44	20	L	F	27298	ANR	73	13	16	11	20.5	24	29	110.4	99.2	98.0	96.3
777	M	51	14	L	F	37677	Tumor	131	26	18	11	26	24	29	117.6	96.6	96.0	93.8
792	F	30	14	L	F	167344	Tumor	23	14.5	14	10	17	24	27	106.1	99.6	-	-
795	F	50	16	L	F	15232	Tumor	60	21.5	18	12	20	24	30	124.8	96.0	100.0	100.0
796	F	48	16	L	T	69419	Tumor	132	12	17	9.5	20.5	24	30	112.8	100.0	98.8	90.0


*BG, basal ganglia; CC, corpus callosum; Cer, cerebellum; CR, corona radiata; F, frontal; Ins, insula; O, occipital; P, parietal; T, temporal; Thal, thalamus; HEM, hemorrhage; Tumor, tumor resection; ANR, aneurysm rupture; Exec, executive function; Mem, episodic memory; VisSp, visuospatial; Lang, language; Beh, behavior/social comportment. ^*1*^ Voxel size = 1 mm × 1 mm × 1 mm; ^*2*^ Within normal limits cut-off = 27; ^*3*^ Within normal limits cut-off = 93.8.*

**Table 2:** Demographic and neuropsychological profiles of RH cases.

ID#	Sex	Age	Education (years)	Lesion Side	Region	Lesion volume^1^	Etiology	Chronicity (months)	PBAC					MMSE^2^	AM-NART	WAB (AQ)^3^	OANB	
													
									Mem	VisSp	Lang	Exec	Beh				Axn	Obj
									(0–27)	(0–18)	(0–12)	(0–26)	(0–24)	(0–30)				
083	M	72	12	R	FTP	8040	Stroke	199	17	16	12	23.5	24	29	114.0	99.8	97.5	96.0
087	F	73	16	R	F	10543	Stroke	192	23.5	17	10	20	24	28	113.0	99.1	-	-
101	F	59	18	R	T/BG	64191	Stroke	449	20	14	12	17.5	24	29	121.0	98.4	98.0	98.0
112	F	50	16	R	O/Thal	4733	Stroke	204	22	18	12	23	24	29	119.0	100.0	100.0	98.0
264	F	67	13	R	F	45305	HEM	212	–	–	–	–	–	–	-	-	-	-
294	F	66	14	R	Ins/BG/CR/Cer	17455	Stroke	175	24	15	12	21.5	22	29	117.0	99.4	100.0	100.0
312	F	63	16	R	P	32649	AVM	397	18	14	12	22.5	24	29.5	-	100.0	97.5	100.0
444	F	81	12	R	PT	15496	Stroke	120	15	13	11.5	21.5	24	25	99.0	95.5	93.0	94.0
474	F	53	11	R	P	22208	Stroke	129	21	12	12	17.5	24	28	89.0	95.1	95.1	98.0
552	F	66	13	R	F	4080	AN	163	18.5	18	12	22	24	30	106.0	99.4	100.0	100.0
569	F	74	18	R	FT	37366	Stroke	98	20.5	17	12	25.5	24	29	125.0	100.0	99.0	99.0
592	F	46	12	R	PT	130552	Stroke	138	19	14	12	19	22	29	110.0	97.8	98.0	98.0
593	F	52	12	R	FTP/BG	170128	Stroke	87	10.5	10	10	15.5	24	27	95.4	100.0	95.0	90.0
640	F	72	18	R	TP	64603	Stroke	76	–	–	–	–	–	–	126.0	96.8	100.0	100.0
657	M	77	18	R	PO	33568	Stroke	66	21	18	12	21.5	24	28	126.2	99.2	100.0	98.0
665	F	54	12	R	P	30092	Tumor	167	22	18	12	22	24	30	110.0	99.2	100.0	98.0
675	M	67	18	R	PT	23779	Stroke	68	20	18	7.5	15	24	29	119.8	99.3	97.5	96.0
694	F	36	12	R	FP/BG/Ins	46499	Stroke	124	15.5	18	9	20.5	24	27	106.0	96.2	97.5	98.0
785	F	60	12	R	F	7718	Tumor	100	–	–	–	–	–	–	-	99.1	-	-
815	F	67	14	R	F	69423	Tumor	208	19	17	12	22	24	30	116.0	98.6	98.8	98.0


*BG, basal ganglia; CC, corpus callosum; Cer, cerebellum; CR, corona radiata; F, frontal; Ins, insula; O, occipital; P, parietal; T, temporal; Thal, thalamus; HEM, hemorrhage; Tumor, tumor resection; ANR, aneurysm rupture; Exec, executive function; Mem, episodic memory; VisSp, visuospatial; Lang, language; Beh, behavior/social comportment. ^*1*^ Voxel size = 1 mm × 1 mm × 1 mm; ^*2*^ Within normal limits cut-off = 27; ^*3*^ Within normal limits cut-off = 93.8.*

Several neuropsychological measures were administered to patients to better characterize their cognitive abilities and deficits. The Mini-Mental Status Exam (MMSE; Folstein et al., 1975) was administered to provide a general impression of cognitive status. The Philadelphia Brief Assessment of Cognition (PBAC; Libon et al., 2011), a cognition-screening instrument, was administered to assess function in five cognitive domains: working memory/executive control, lexical retrieval/language, visuospatial/visuo-constructional operations, verbal/visual episodic memory, and behavior/social comportment. Given the verbal nature of the study, WAB (Kertesz, 1982) was administered to better characterize language comprehension and production abilities, and the American version of the Nelson Adult Reading Test (AMNART; Blaire and Spreen, 1989) was administered to establish an estimate of premorbid verbal IQ. Since nouns and verbs comprised the critical figuratively extended words in our metaphors, the Object and Action Naming Battery (OANB; Druks, 2000) was also administered to specifically assess lexical access for common object and action names. Although it was not possible to collect all of these measures on every single patient, independent samples *t*-tests on available scores indicated LH and RH patients had similar language abilities. LH patients did not differ significantly from RH patients on the MMSE, OANB, AMNART or PBAC Total Score. Further, though LH WAB performance indicated a significantly lower Aphasia Quotient [*t*(36) = -2.15, *p* = 0.04], the difference was small (LH = 94.7, *SD* = 9.7; RH = 98.6, *SD* = 1.6), and both groups were considered within normal limits.

Twenty neurologically healthy older adults recruited from the Center for Cognitive Neuroscience Normal Control Database served as a control population (Age: 63.8 ± 8.7, Education: 14.3 ± 2.5) and were paid $15/h for their participation. All participants were native English speakers, right-handed, and gave informed consent to participate in accordance with the Institutional Review Board of the University of Pennsylvania (data from 12 of these participants was published previously in our related paper; Ianni et al., 2014). Controls did not differ significantly from patients in age or education.

### Stimuli

#### Sentences

Stimuli consisted of 60 literal-metaphor sentence pairs of three types: nominal-entity, nominal-event, and predicate. Both nominal sentence types were category assertions of the form “*The X was a Y*,” with 1–2 modifying adjectives. Predicate items consisted of a noun phrase and an action verb followed by a prepositional phrase, with 1–2 adjectives modifying the agent or patient of the sentence. In nominal-entity items, nouns referring to concrete entities or objects (e.g., *bullet, cheetah, drum*) served as the metaphorically extended source words. In nominal-event items, nominalized verbs were extended metaphorically [e.g., *(a) dance, (a) limp, (a) fall*]. In predicate items, action verbs were extended metaphorically (e.g., *ran, giggled, argued*).

These 60 literal-metaphor sentence pairs (20 nominal-entity, 20 nominal-event, and 20 predicate) were selected from a superset of 400 sentence pairs (40 pairs were taken from Cardillo et al., 2010 and 20 pairs from Cardillo et al., 2017). Item selection using Stochastic Optimization of Stimuli (SOS) software (Armstrong et al., 2012) ensured metaphors and literals (within and across each sentence type) were matched in terms of familiarity, length (number of words, number of content words, number of characters), average content word frequency, average content word concreteness, and positive valence ratio (*p*’s > 0.10). As previously observed (Cardillo et al., 2010, 2017), metaphors were judged to be significantly less imageable (*p* < 0.005) and natural (*p* < 0.01) than their literal counterparts, and significantly more figurative (*p* < 0.005). Selection criteria further ensured sentences of different types (nominal-entity, nominal-event, predicate) were also matched on interpretability (metaphors only), figurativeness (metaphors only), familiarity, naturalness, imageability, length (number of words, number of content words, number of characters), frequency, concreteness, and positive valence ratio (*p*’s > 0.10). The sensory modality of source terms was not manipulated in this stimulus set; nonetheless, we used SOS to ensure that auditory and visual imagery of base terms did not differ across sentence types. Note: Valence RT did not differ significantly across metaphors and literals of each sentence type, but did differ significantly in one comparison between sentence types: Nominal-Entity items were faster than Predicate items (*p* < 0.05). Means and standard deviations of these psycholinguistic variables are summarized in Table 3.

**Table 3:** Psycholinguistic properties of literal and metaphoric sentences (reproduced from Ianni et al., 2014).

	Literal			Metaphor		
		
	Nominal-Entity	Nominal-Event	Predicate	Nominal-Entity	Nominal-Event	Predicate
		
	*M (SD)*	*M (SD)*	*M (SD)*	*M (SD)*	*M (SD)*	*M (SD)*
Base auditory imagery	2.63 (1.2)	2.61 (1.4)	2.07 (1.16)	2.63 (1.2)	2.61 (1.4)	2.07 (1.16)
Base visual imagery	3.66 (1.14)	3.2 (0.59)	3.41 (0.72)	3.66 (1.14)	3.2 (0.59)	3.41 (0.72)
Concreteness	480 (76)	474 (46)	500 (53)	450 (57)	449 (69)	474 (76)
Frequency^∗^	92.9 (159)	89.9 (142.4)	86.7 (85.3)	90.8 (123.7)	91.8 (128)	95.6 (133.7)
No. of characters	33.3 (4.2)	32 (5.1)	33.6 (5.2)	34.3 (4.6)	32.7 (5.2)	34.9 (4)
No. of words	6.1 (0.4)	6.2 (0.4)	6.2 (0.5)	6.1 (0.6)	6.1 (0.5)	6 (0.6)
No. of content words	3.2 (0.5)	3.2 (0.4)	3.3 (0.5)	3.2 (0.5)	3.1 (0.5)	3.3 (0.4)
Interpretability	n/a	n/a	n/a	0.94 (0.08)	0.94 (0.08)	0.96 (0.05)
Familiarity	5.28 (0.73)	5.14 (1.11)	5.26 (1.23)	4.96 (0.76)	4.83 (1.18)	4.86 (1.37)
Naturalness	5.68 (0.73)	5.76 (0.95)	5.48 (1.24)	4.84 (0.82)	5.1 (1.07)	4.8 (1.34)
Imageability	5.55 (0.83)	5.67 (0.97)	5.8 (1.08)	4.17 (0.97)	4.27 (0.78)	3.94 (1.16)
Figurativeness	1.88 (0.73)	2.02 (0.92)	1.78 (0.91)	5.62 (0.56)	5.28 (0.77)	5.25 (1.02)
Valence RT	1279 (213)	1390 (182)	1426 (237)	1351 (131)	1432 (220)	1495 (200)


*^∗^SUBTL_*WF*_ values from Brysbaert and New (2009).*

#### Answer Choices

Four answer choices were generated to accompany each sentence: one correct target and three incorrect foils. All answer choices were composed of an adjective followed by a noun. In the metaphor condition, the target expressed the (figurative) meaning of the sentence, Foil 1 expressed the literal sense of the sentence, Foil 2 expressed the opposite of the (metaphorical) meaning of the sentence, and Foil 3 was unrelated to the sentence meaning. Foils were designed to be informative of the type of language deficit present. A Foil 1 selection indicates a literal bias in metaphor comprehension. A Foil 2 selection indicates impaired semantic integration, as the metaphorical sense of the source word was necessarily activated but incorrectly interpreted in the context of the sentence. A Foil 3 selection indicates a more general and profound comprehension deficit, as it has no relation to the sentence.

In the literal condition, the foils were designed to mirror the difficulty and nature of foil types in the metaphor condition as closely as possible. The target expressed the (literal) meaning of the sentence, Foil 1 was related to the agent of the sentence by category membership (but not implied by the sentence), Foil 2 expressed the opposite of the (literal) meaning of the sentence, and Foil 3 was unrelated to the sentence meaning. Thus, Target, Foil 2, and Foil 3 were the same as in the Metaphor condition. Because it was not possible to have the same kind of answer for Foil 1 across Metaphor and Literal conditions, Foil 1 for literal sentences were designed to mirror the lexical-semantic selection demands of Foil 1 answers in the metaphor condition (i.e., both present a meaning strongly associated with the source term). Given the reversed valence necessarily entailed by the Foil 2 condition (the opposite of the target meaning), an additional constraint on all answer choices was introduced to avoid valence-related biases in selection: for both metaphor and literal items, Target and Foil 2 had opposite valences and Target and Foil 3 had the same valence.

To avoid inadvertent difficulty differences across answer choices, we also gathered frequency and concreteness values for the individual words making up each answer choice. Frequency values were collected from SUBTLEXus (Brysbaert and New, 2009) and concreteness values were collected from the MRC Psycholinguistic Database (Coltheart, 1981) and the University of South Florida Norms (Nelson et al., 2004). For those words that did not have published concreteness values, we collected our own using the procedures of Cardillo et al. (2010). For comparing across conditions, the values for each answer choice were averaged in order to generate a single frequency and concreteness value per answer choice.

Independent *t*-tests indicated no significant differences in average frequency between sentence types or between answer choice types within and across literal and metaphor conditions. Unsurprisingly, given the abstract nature of metaphor, Target and Foil 1 answer choices in the metaphor condition did significantly differ in terms of average concreteness (*p* < 0.005). To avoid any concreteness-related bias in selecting answers, we contrived an additional constraint on all answer choices: we modified our answer choices so that Target and Foil 3 also significantly differed in concreteness (*p* < 0.005) but Target and Foil 2 did not (*p* > 0.10). We modified our literal answer choices as well so that they, too, followed this pattern: Target and Foil 1 differed in concreteness (*p* < 0.001), as did Target and Foil 3 (*p* < 0.005), but Target and Foil 2 did not (*p* > 0.10). As such, our final set of answer choices were matched on frequency, concreteness, and valence so none could aid blind guessing. Table 4 provides examples of sentence and answer choice stimuli. Full materials are available upon request.

**Table 4:** Sentence and answer choice examples (reproduced from Ianni et al., 2014).

Sentence	Type	Example	Target	Foil 1	Foil 2	Foil 3
Metaphor	Nominal-Entity	The coffee was a caffeine bullet.	Energy jolt	Military ammunition	Soothing lullaby	Funny teacher
	Nominal-Event	His interest was a mere sniff.	Weak enthusiasm	Runny nose	Delighted fascination	Rotten fruit
	Predicate	The debate spun into a brawl.	Violent incident	Twirling form	Peaceful resolution	Toxic fumes
Literal	Nominal-Entity	The police evidence was a bullet.	Lethal weapon	Confiscated goods	Hospital bandage	Circus tent
	Nominal-Event	The rabbit’s twitch was a sniff.	Nose wiggle	Epileptic fit	Completely motionless	Yoga class
	Predicate	The top spun into the box.	Whirling motion	Glass marble	Fixed position	Tiny sailboat


### Procedure

#### Control Procedure

All participants made judgments on all items. Subjects were instructed to choose the answer choice that best matched the “meaning of the sentence,” and to guess if unsure. Participants pressed the space bar once for the sentence to appear, and a second time to view its answer choices. Answer choices were presented in quadrant format below the sentence and remained on the screen until a response was selected using one of four keys on the keyboard. Sentences were presented centrally in black, 18-point font on a white background using E-Prime 1.1 software on a Dell Inspiron laptop. Each participant received a unique, random order of items. The target and each foil had a 25% chance of appearing in any single quadrant on the screen in any given trial. Ten practice trials preceded four blocks of experimental trials.

#### Patient Procedure

The only difference between Control and Patient tasks was a change from self-paced to experimenter-advanced trials to avoid memory or motor response difficulties. In the patient version of the task, the experimenter pressed the spacebar to prompt the appearance of the sentence. After a 3-s delay, the answer choices were presented beneath the sentence. Patients indicated their choice by pointing to or saying the answer aloud and the experimenter recorded their selection using the keyboard.

### Analysis

An item analysis of healthy controls’ scores revealed three items whose comprehension fell three standard deviations below the average; these items were eliminated from further analysis. A subject analysis of accuracy scores revealed a single individual whose comprehension fell three standard deviations below average on any given sentence-type; this individual was replaced. For controls, accuracy for literal and metaphor conditions was averaged across all participants. For patients, accuracy in the literal and metaphor conditions was averaged across all patients for the Group analyses and calculated separately for each individual for the Single Case analyses. Foil profiles were generated for each patient by dividing the number of each type of error (Foil 1, Foil 2, Foil 3) by the total number of errors in literal and metaphor conditions. Raw data supporting the conclusions of this manuscript are available from the authors on request.

#### Group Analysis

To compare healthy and brain-injured populations, a three-way omnibus ANOVA of Figurativeness (Metaphor, Literal) × Sentence Type (Nominal-Entity, Nominal-Event, Predicate) × Group (Controls, LH, RH) was calculated on accuracy per subject per condition. To better understand this pattern of effects and to specifically address our hypotheses about laterality, this ANOVA was further broken down into two, more targeted analyses:

For Controls, a two-way, within-subjects ANOVA of Figurativeness (Metaphor, Literal) × Sentence Type (Nominal-Entity, Nominal-Event, Predicate) was calculated on accuracy per subject per condition.

For Patients, a three-way, mixed ANOVA of Figurativeness (Metaphor, Literal) × Sentence Type (Nominal-Entity, Nominal-Event, Predicate) × Group (LH, RH) was calculated on accuracy per subject per condition.

#### Single Case Analysis

As in Ianni et al. (2014), we tested for three patterns of deficit: a general deficit (impaired comprehension for both literal and metaphoric sentences); a selective deficit (impaired comprehension for metaphoric sentences only), and a differential deficit (a larger deficit in metaphoric than literal sentences). We used a “Bayesian analysis for a *simple* difference,” developed by Crawford et al. (2010) to test for comprehension deficits affecting either the literal or metaphoric conditions. This test uses Bayesian Monte Carlo methods to determine if a patient’s score is sufficiently below the scores of controls such that the null hypothesis, that the patient’s score is an observation from the control population, can be rejected. In this case, patients with a *simple* metaphor or literal deficit exhibit significantly reduced comprehension in that condition, relative to controls.

We used a “Bayesian analysis for a *differential* difference,” also developed by Crawford et al. (2010), to test for a differential deficit in metaphor comprehension at the level of the individual patient. Following the logic and proposal of Crawford et al. (2003), we maintain that the observation of simple deficits is necessary but not sufficient for asserting a dissociation between two cognitive functions. To demonstrate a “classical” dissociation requires that three criteria be met: (1) a patent’s performance on Task X meets the criteria for a deficit relative to healthy controls, (2) that same patient’s performance on Task Y is within normal limits and fails to meet deficit criteria, and (3) performance on Task X is significantly worse than on Task Y. Demonstration of a “strong” dissociation requires that both Tasks X and Task Y meet criteria for a deficit, and that performance on one task is significantly worse than for the other.

With respect to the current study, the Bayesian test for a *simple* difference can only indicate whether a patient is impaired in the metaphor, literal, or both conditions. It does not distinguish between reduced accuracy due to difficulty with metaphor specifically and reduced accuracy due to a general impairment affecting literal and metaphoric language alike. The Bayesian test for a *differential* difference, however, can make this distinction by also taking into account the difference between an individual case’s metaphor and literal accuracy, the literal-metaphor accuracy difference observed in the Control group, and the correlation between the two conditions, as established by the Control group. Patients with a *differential* metaphor deficit exhibit greater difficulty with metaphoric than literal sentences than is observed in the control population. Without the differential difference test, we cannot be confident two tasks truly dissociate; without the simple difference tests, we cannot know whether the difference between the tasks is a trivial one or reflects genuinely impaired cognition. Both simple and differential tests were calculated using Bayesian criteria, *z*-scores, 1-tailed significance testing, and a calibrated prior in the DissocBayes_ES software (Crawford et al., 2010).

We also used single case statistics to consider the possibility that comprehension of different types of metaphors can be selectively impaired. To test this hypothesis, we applied the Bayesian analysis for a differential difference separately to each sentence type (Nominal-Entity, Nominal-Event, and Predicate) for each patient showing difficulty specific to metaphors.

## Results

### Group Analysis

#### Omnibus ANOVA

The three-way ANOVA of Figurativeness (Metaphor, Literal) × Sentence Type (Nominal-Entity, Nominal-Event, Predicate) × Group (Controls, LH, RH) revealed significant main effects of Figurativeness [*F*(1,19) = 22.640, *p* = 0.0005, ε^2^ = 0.284] and Sentence Type [*F*(2,57) = 17.637, *p* = 0.0005, ε^2^ = 0.236], and a significant interaction of Figurativeness × Type [*F*(2,76) = 8.599, *p* = 0.0005, ε^2^ = 0.131], indicating the impact of figurativeness on comprehension accuracy varied across the different syntactic forms tested. The main effect of Group approached significance [*F*(2,57) = 2.898, *p* = 0.063, ε^2^ = 0.092], reflecting higher overall accuracy in Controls than RH patients, and RH patients than LH patients (Control *M* = 91.7, *SD* = 0.08; RH M = 90.4, *SD* = 0.11, LH *M* = 84.1, *SD* = 0.13). Although the three-way interaction of Figurativeness × Type × Group was not significant, we further broke down this ANOVA into separate analyses for healthy and brain-injured patients to better characterize these patterns and to maximize our ability to test our hypotheses about laterality. Mean accuracy, broken down by condition and group, is reported in Table 5.

**Table 5:** Mean accuracy by condition and group.

	Literal			Metaphor		
		
	Nominal-Entity	Nominal-Event	Predicate	Nominal-Entity	Nominal-Event	Predicate
		
	*M (SD)*	*M (SD)*	*M (SD)*	*M (SD)*	*M (SD)*	*M (SD)*
Controls	92.0 (10.2)	94.5 (8.4)	94.0 (5.5)	88.0 (10.1)	90.0 (11.7)	92.3 (9.5)
RH	92.8 (10.0)	92.3 (12.2)	95.3 (6.2)	82.3 (22.5)	89.1 (12.4)	93.1 (10.2)
LH	87.5 (11.2)	87.8 (15.9)	90.5 (14.1)	72.3 (22.6)	79.3 (21.1)	87.8 (10.1)


#### Controls

Overall, Controls performed well on the task (*M* = 91.7, *SD* = 7.7). Accuracy was positively correlated with subjects’ years of education (*R* = 0.515, *p* = 0.02), but not with their age. Accuracy in Literal and Metaphoric conditions was also significantly correlated (*R* = 0.826; *p* < 0.0005).

**FIGURE 2:**
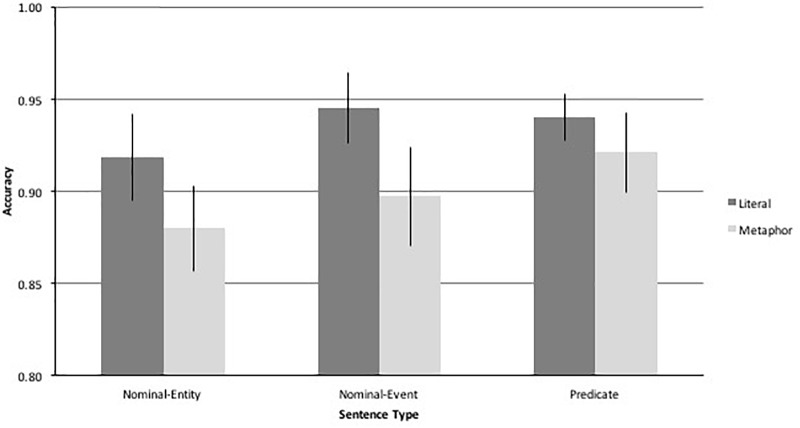
Control accuracy with metaphoric and literal sentences of different types.

The two-way ANOVA of Figurativeness (Metaphor, Literal) × Sentence Type (Nominal-Entity, Nominal-Event, Predicate) revealed a significant main effect of Figurativeness [*F*(1,19) = 9.128, *p* = 0.007, ε^2^ = 0.325], reflecting higher accuracy in the literal (*M* = 93.5, *SD* = 7.0) than metaphoric condition (*M* = 89.9, *SD* = 9.2). The main effect of Sentence Type approached significance [*F*(2,38) = 2.793, *p* = 0.074, ε^2^ = 0.128], suggesting the three syntactic constructions differed in difficulty. The interaction of Figurativeness × Sentence Type was not significant, indicating that the difficulty difference associated with Sentence Type was similar for literal and figurative sentences. A linear contrast test of Sentence Type indicated a significant linear gradient of difficulty across conditions: Nominal-Entity < Nominal-Event < Predicate [*F*(1,19) = 4.826, *p* < 0.041, ε^2^ = 0.203]. This pattern is plotted in Figure 2.

Although performance was generally high, answer choice selection on incorrect trials illuminated the nature of Controls’ occasional misunderstandings. In the metaphor condition, Foil 1 (the literal sense of the sentence) was the most common error (65.6%), followed by Foil 2 (28.2%) and Foil 3 (6.3%). Likewise, in the literal condition, Foil 1 (related to the agent of the sentence by category membership, but not implied by the sentence) was the most common error (64.9%), followed by Foil 2 (28.2%) and Foil 3 (6.9%).

#### Patients

Overall, Patients performed modestly worse than Controls on the task (*M* = 87.5, *SD* = 12.4). Like Controls, Patient accuracy was positively correlated with years of education (*R* = 0.369, *p* = 0.02), but not with age, lesion volume, or lesion chronicity. Patient accuracy in Literal and Metaphoric conditions was also strongly correlated (*R* = 0.709; *p* < 0.0005).

The three-way ANOVA of Figurativeness (Metaphor, Literal) × Sentence Type (Nominal-Entity, Nominal-Event, Predicate) × Group (LH, RH) revealed a significant main effect of Figurativeness [*F*(1,38) = 15.74, *p* < 0.005, ε^2^ = 0.0293], reflecting higher accuracy in the literal (*M* = 91.0, *SD* = 10.8) than metaphoric condition (*M* = 84.0, *SD* = 16.0). The main effect of Sentence Type was also significant [*F*(2,76) = 15.33, *p* < 0.0005, ε^2^ = 0.288], indicating the three syntactic constructions differed in difficulty. A linear contrast test of Sentence Type indicated a significant linear gradient of difficulty across conditions: Nominal-Entity < Nominal-Event < Predicate [*F*(1,38) = 27.66, *p* < 0.0005, ε^2^ = 0.421]. The interaction of Figurativeness × Type was also significant [*F*(2,76) = 10.17, *p* < 0.0005, ε^2^ = 0.211], indicating that the effect of Sentence Type differed in Metaphor and Literal conditions. The main effect of Group was not significant, nor were any of its interactions, indicating LH and RH patients responded to the task similarly.

**FIGURE 3:**
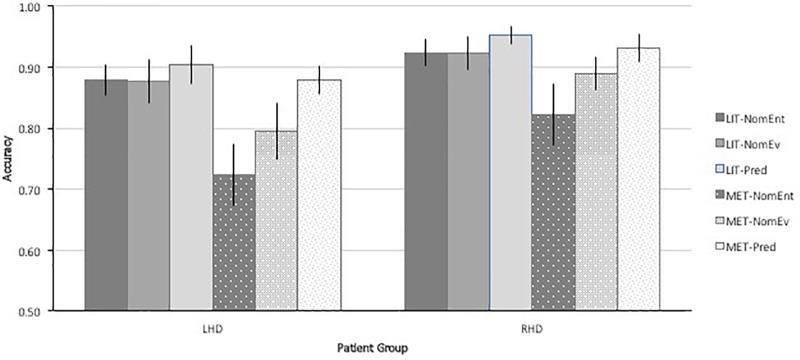
Patient accuracy with metaphoric and literal sentences of different types.

To better understand the pattern of results, the significant interaction of Figurativeness × Sentence Type is plotted in Figure 3, separately for LH and RH patients. The pattern reveals accuracy differed by sentence type only in the metaphoric conditions.

Answer choice selection on incorrect trials revealed that while patients made more errors than healthy controls, the nature of their misunderstandings followed a similar pattern in the metaphor condition. Foil 1 (the literal sense of the sentence) was the most common error (62.7%), followed by Foil 2 (25.7%) and Foil 3 (3.4%). Unlike Controls, in the literal condition, patients were equally likely to select Foil 1 (related to the agent of the sentence by category membership, but not implied by the sentence, 44.5%) as Foil 2 (48.2%), and unlikely to select Foil 3 (2.2%).

### Single Case Analysis

Application of the Bayesian test for a simple deficit revealed a simple metaphor comprehension deficit in seven patients. Six of these cases were LH patients (360, 384, 493, 529, 642, 729) and one was a RH patient (593). Of these seven cases, two also presented with a simple literal comprehension deficit (360, 593). The Bayesian test for a differential deficit was applied to these seven cases in order to distinguish three deficit patterns of interest: (1) a *General Comprehension Deficit*, in which both literal and metaphor comprehension are impaired, but not differentially, (2) a *Differential Metaphor Deficit* (i.e., Strong Dissociation), in which both metaphor and literal comprehension is impaired, but metaphor more so, and (3) a *Selective Metaphor Deficit* (i.e., Classical Dissociation), in which metaphor comprehension is impaired but literal comprehension is spared. Following the suggestion of Crawford et al. (2003), we corrected for multiple comparisons at this level, adjusting our alpha-criterion using the Bonferroni method.

**FIGURE 4:**
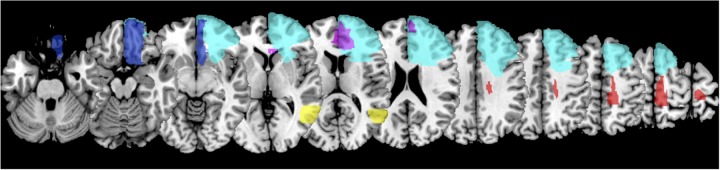
Overlay map depicting lesions of patients with metaphor but not literal deficits, presented in MNI space and following radiological convention (left side = right hemisphere).

Results indicated two cases qualified as having a General Comprehension Deficit (360, 593), no cases met the criteria for a Differential Metaphor Deficit/Strong Dissociation, and four cases met the criteria for a Selective Metaphor Deficit/Classical Dissociation (384, 493, 529, 642). One case exhibiting a simple metaphor deficit (792) failed to meet the criteria for either a Classical or Strong Dissociation (i.e., they were impaired on metaphors, but the difference between their Literal and Metaphor accuracies was not larger than one might expect to observe in the Control population). Behavior of these cases is summarized in Table 6. To better appreciate the critical brain areas implicated for metaphor comprehension, a lesion overlay for the four selective metaphor deficit cases and single borderline case (792) is presented in Figure 4. See Supplementary Table 1 for detailed reporting of the statistics associated with each case.

**Table 6:** Behavioral profile of cases with impaired metaphor comprehension.

Patient ID	Literal accuracy	Metaphor accuracy	Simple metaphor deficit	Simple literal deficit	Differential metaphor deficit	Classification	Literal errors			Metaphor errors		
								
							F1	F2	F3	F1	F2	F3
360	42.4	43.1	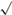	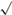	x	General	0.61	0.24	0.15	0.58	0.27	0.15
593	64.4	36.2	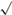	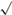	x	General	0.38	0.48	0.14	0.73	0.19	0.08
384	88.1	51.7	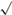	x	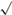	Selective	0.14	0.86	0.00	0.93	0.07	0.00
493	94.9	60.3	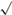	x	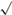	Selective	0.00	1.00	0.00	0.74	0.22	0.04
529	86.4	58.6	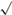	x	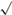	Selective	0.38	0.50	0.13	0.77	0.15	0.08
642	94.9	65.5	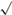	x	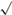	Selective	0.33	0.67	0.00	0.57	0.33	0.10
792	81.4	69.0	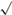	x	x	–	0.33	0.58	0.08	0.75	0.25	0.00


To test for the possibility that metaphor impairments can be specific to different types of metaphor, we also applied the Bayesian tests for a differential deficit to each sentence type separately for each of the five Metaphor-impaired patients identified in the previous analysis. Results revealed distinct deficit patterns that were obscured in the previous analysis when averaging across metaphor types: two patients exhibited a metaphor deficit impairing nominal metaphor comprehension but sparing predicate metaphor comprehension (384, 493), one patient exhibited a metaphor impairment affecting nominal-event and predicate metaphors (642), and one patient exhibited an impairment effecting only Nominal-Entity metaphors (529), and one patient showed a more complex patterns suggestive of difficulty with nominal metaphors and/or syntax, but failed to meet criteria for a dissociation (792). These patterns of performance by sentence type are summarized in Table 7. See Supplementary Table 2 for detailed reporting of these statistics for each case.

**Table 7:** Behavioral profiles of cases with metaphor impairments that differ by sentence type.

Patient ID	Nominal-Entity		Deficits			Nominal-Event		Deficits			Predicate		Deficits			Nature of metaphor deficit
							
	Lit Acc	Met Acc	Simple Met	Simple Lit	Differential	Lit Acc	Met Acc	Simple Met	Simple Lit	Differential	Lit Acc	Met Acc	Simple Met	Simple Lit	Differential	
384	84.2	35.0	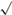	x	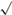	85.0	36.8	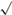	x	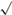	95.0	84.2	x	x	x	Nominals
493	94.7	50.0	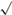	x	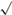	95.0	47.4	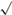	x	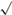	95.0	84.2	x	x	x	Nominals
529	78.9	30.0	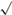	x	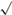	95.0	73.7	x	x	x	85.0	73.7	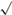	x	x	Nominal-Entity
642	89.5	65.5	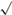	x	x	95.0	68.4	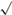	x	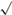	100.0	68.4	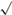	x	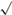	Nominal-Event + Predicates
792	68.4	55.0	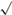	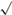	x	80.0	68.4	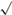	x	x	95.0	84.2	x	x	x	–


## Discussion

Given the prominence of metaphor in human cognition and speech, the current study sought to shed light on the outstanding ambiguity concerning its neural substrates. To do so, we tested a large group of focal lesion patients with unilateral brain injury on a metaphor comprehension task and compared their individual performances to the behavior of a group of age- and education-matched healthy control subjects. Our results reveal three major findings: (1) metaphor comprehension can be selectively impaired after brain injury, (2) damage to the left, not right, hemisphere produces selective impairments understanding metaphors, and (3) different types of metaphors place different cognitive and neural demands.

Although nothing in their clinical records indicated a reason to suspect difficulty with metaphors or non-literal language more generally, seven of the 40 patients we tested were significantly less accurate selecting the meaning of metaphoric sentences than the behavior of the control group indicates is normal. Two of these patients (360, 593) were comparably impaired identifying the meaning of literal sentences, consistent with a *General Comprehension Impairment* rather than a figurative one. These two patients are outliers compared to the rest of the patients in two ways. Patient 360, a left temporal lobe stroke survivor, is the only patient in the cohort with marked aphasia (Wernicke’s classification based on WAB). Neuropsychological testing for all other patients revealed Aphasia Quotients > 90 on the WAB and >90% accuracy on the Objects and Actions subtests of the OANB. By contrast, Patient 360’s Aphasia Quotient was 65.3 and his OANB naming accuracy was also extreme (Objects, 28%; Actions, 52%). His error profile was similar for metaphor and literal conditions: approximately 60% Foil 1, 25% Foil 2, and 15% Foil 3, indicating a bias to select strongly associated but contextually irrelevant meanings. Patient 593 – the single RH patient to show any difficulty with the task – was exceptional in a different way: she had suffered the largest brain injury of any patient, surviving a large right fronto-temporal-parietal stroke that extended to the right basal ganglia and caudate nucleus. Her language skills were normal, as indexed by a WAB Aphasia Quotient of 100 and high accuracy on the OANB, but her PBAC performance indicated impaired executive function, memory, and visuospatial processing. Her error pattern differed across conditions: when interpreting metaphors, she showed a literal bias (73% Foil 1 selection), but when reading literal sentences, she was equally likely to choose Foil 1 (category associate) as Foil 2 (opposite meaning). It is not possible to determine precisely the nature of the comprehension difficulties of these two patients without further testing, but based on the nature of their other deficits and error profiles, it seems likely that they are dissimilar in nature.

Of the five other patients that exhibited abnormally low accuracy on the metaphor condition, four of them met formal criteria for a *Selective Metaphor Impairment* (i.e., a dissociation, putatively classical; Crawford et al., 2003). That is, they performed normally on the literal sentences and the magnitude of their accuracy difference between metaphoric and literal conditions was larger than expected based on performance of the control group. The fifth patient met only one of these two additional criteria, suggesting a mild difficulty with metaphor but not a true dissociation with literal comprehension. One of the five patients (384) exhibited mild word finding difficulty, as reflected by his WAB Aphasia Quotient of 90.8, but the language skills of this set of patients were otherwise normal. The finding here of selective metaphor impairments in the absence of any known comprehension deficits accords with our earlier case-series report involving a smaller control group (Ianni et al., 2014)^1^.

What is most striking about the current dataset is that none of the patients exhibiting selective metaphor impairments had injuries affecting the right hemisphere. This failure to support either formulation of the RH hypothesis was obtained despite testing RH patients with lesions in the areas with the greatest probability of resulting in a deficit. Previous lesion studies have not included sufficient neuroanatomical detail to indicate which specific regions of the right hemisphere might be critical for metaphor. However, pointers are provided by other methodologies. Pobric et al. (2008) used transcranial magnetic stimulation (TMS) to test for RH involvement in metaphor processing, with results suggesting the right superior temporal sulcus might be an important area. On the other hand, right inferior prefrontal cortex is the RH area most reliably activated by imaging studies (Bohrn et al., 2012; Rapp et al., 2012). Both of these regions were well-represented in the distribution of lesions affecting our RH patients, but patients with injury to either of these areas failed to demonstrate selective difficulty with metaphors. One limitation of our study is that the distribution of lesions in our sample left us unable to test the contribution of some cortical areas (e.g., superior parietal cortex in the LH and inferior and anterior temporal cortex in the RH; see Figure 1). The gaps in our coverage do not impact areas generally implicated by previous patient and neuroimaging research on metaphor; nevertheless, it’s possible they are overlooked but necessary areas, or bilateral damage to these areas is required to observe deficits. Although not well-supported by existing research, our current sample of patients cannot rule out these possibilities for RH necessity.

Although our findings contradict the observations of previous metaphor studies with RH focal lesion patients, those earlier data are more equivocal than they are often taken to be. In their seminal study, Winner and Gardner (1977) found that the same RH patients who expressed a bias for a literal interpretation of metaphors when assessed with a picture-matching task, showed intact comprehension when asked to provide verbal explanations of those metaphors. Similarly, Giora et al. (2000) found no significant difference between RH patients and controls using an oral explanation task, and Zaidel et al. (2002) observed no significant differences between LH and RH performance on verbal and pictorial assessments of metaphor comprehension once visuo-spatial and linguistic deficits were included as covariates in their analyses. The task dependence of RH metaphor deficits is frequently noted, but less often is its implication: such task dependence calls into question any necessary or privileged role for the RH in metaphor.

Other problematic details for the RH hypothesis for metaphor also seem relevant when viewed with a skeptical lens. Brownell et al. (1984) found RH patients selected the metaphoric meaning of a polysemous word less often than LH patients – but excluded from analyses RH patients who performed at ceiling, effectively amplifying the odds of detecting a group effect. Giora et al. (2000) reported no significant difference between RH patients and controls, as did Klepousniotou and Baum (2005a), and MacKenzie et al. (1999) reported no significant difference between RH patients and controls when they were older ages (>75 years). The latter study did observe significantly lower accuracy relative to controls on their metaphor-picture matching task when RH patients and controls were younger (<75 years), but their patient population was unusual in that they were only 1 month post-stroke. All other lesion studies have considered chronic patients (>6 months post-stroke). Testing patients so soon in their recovery process, before restoration and reorganization has occurred, has its own clinical and theoretical value, but will capture difficulties that later resolve and presents a misleading picture when lumped together with chronic patients.

On the whole, either version of the RH hypothesis of metaphor does not hold up well under empirical scrutiny, whether considering it through the lens of neuroimaging or lesion studies. We interpret the inconsistent, weak, and/or task-dependent observations to be most consistent with proposals that the RH plays a supportive but non-necessary role whenever language stimuli are complex and demanding to process. This interpretation has been articulated in broad strokes in the Cognitive Resource Hypothesis (Tompkins, 2012), which anticipates greater RH engagement whenever language tasks place greater demands on attention or working memory. In support of this view, Monetta et al. (2006) replicated a RH patient pattern of processing for the alternative metaphorical meanings of words using a dual-task paradigm in healthy participants. More commonly, this flavor of interpretation of RH engagement is evoked in the context of neuroimaging studies of healthy individuals doing more difficult or complex language tasks (e.g., Yang et al., 2009; Prat et al., 2011; Lai et al., 2015), or older adults doing tasks made more taxing as a result of cognitive aging (Li et al., 2001; Cabeza, 2002). For instance, according to the “Dynamic RH Spillover Hypothesis” of Prat et al. (2011), the right hemisphere is not recruited in these more demanding conditions for a particular RH-specific process, but rather, because the attentional and working memory resources of the left hemisphere are sufficiently taxed that processing spills over to exploit the available, but less efficient reserves of RH homologs.

We interpret our failure to find RH impairments in our metaphor multiple choice task to reflect our avoidance of visuospatial task demands that disproportionately impact RH patients, and our careful norming of task and stimuli to ensure our metaphoric and literal conditions were closely matched on psycholinguistic variables affecting comprehension difficulty (e.g., frequency, concreteness, length, valence, familiarity, and a reaction time measure of semantic processing). It is worth noting, however, that we were not able to balance our metaphor and literal conditions on all available measures characterizing our items. Specifically, healthy young adults rated our sample of metaphors as less imageable and natural-sounding than our literal sentences although there is no reason metaphors necessarily be so. One or both of these inadvertent differences may have contributed to the slightly lower metaphor accuracy observed in our older controls when doing our multiple choice task and point to the difficulty of completely equating semantically complex sentences.

Although, we favor an explanation of our null RH results in terms of methodological rigor, our findings are also compatible with the Graded Salience Hypothesis (GSH). In this view, when the meaning of an expression is low in salience – whether because it is unexpected, poorly supported by context, entails subordinate senses of a word(s), or some combination of these factors – the RH is engaged in order to facilitate its access (Giora, 1997). The metaphors in the current task were neither wholly novel, nor highly familiar. Averaging 4.88 on a 7-point familiarity scale, they may be best described as “moderately” or “somewhat” familiar. It is possible that the meanings of our metaphors were sufficiently salient to not require additional RH activity for their comprehension, consistent with the GSH. Follow-up studies with truly novel metaphoric sentences are required to clarify which interpretation is most plausible.

Regardless of the theoretical implication of the present study, the practical implication is clear: LHD should be evaluated for metaphor impairments, even if they do not present with literal comprehension deficits. Given that these impairments have to date flown under the radar of LH neuropsychological evaluation, their social and communicative consequences are wholly unknown. An important issue for future research will be to determine whether and how these impairments might impact the daily lives of chronic LH patients, and what compensatory strategies or therapies effectively ameliorate them.

Another important area for future research is to determine the functional roles of the areas frequently implicated in the metaphor network. Metaphor comprehension is a cognitively complex task. As such, it is likely to rely on a neurally distributed network of brain regions, and comprehension failures could arise from damage to any of these areas or the pathways connecting them. In the current study, four of the five metaphor-impaired patients had lesions restricted to the left frontal lobe. One patient had a lesion injuring left posterior temporal-parietal cortex. Although limited inferences about neural substrates should be made on the basis of single cases, the cases reported here are consistent with the important role of left frontal cortex and posterior temporal cortex for metaphor suggested by others and confirmed in meta-analyses. The left inferior prefrontal cortex is the single most reliably activated brain area in neuroimaging studies of metaphor comprehension, but other left frontal areas like middle frontal gyrus and medial frontal gyrus are also commonly observed (Bohrn et al., 2012; Rapp et al., 2012; Yang, 2014). In a large lesion study of left and right hemisphere patients, Giora et al. (2000) reported a negative correlation between extent of injury in the left temporal-occipital junction and accuracy in a metaphor-picture matching task. Similarly, Zaidel et al. (2002) observed negative correlations between left temporal and temporal-parietal junction injury and accuracy in pictoral and verbal metaphor comprehension tasks, respectively. Meta-analyses, too, implicate the left lateral middle and superior temporal cortices (Bohrn et al., 2012; Rapp et al., 2012; Yang, 2014). Our study also makes the novel suggestion of an important role for ventromedial prefrontal cortex, an area not typically associated with language processing though some studies suggest a role processing other forms of figurative language (Zald and Andreotti, 2010).

The importance of left prefrontal cortex, posterior middle temporal gyrus, and the temporal-parietal junction for the semantic processing of literal language is already well-established (Binder et al., 2009; Noonan et al., 2013). Our results add to a growing body of evidence that suggests metaphor comprehension relies on the same left-hemisphere dominant perisylvian network as literal language does. The seeming absence of unique neural substrates necessary for metaphor comprehension makes it more difficult to argue for the specialness of figurative comprehension processes. Future tasks targeting the possible functional roles of key areas in the metaphor network are necessary to more clearly understand their contribution to metaphor vis-à-vis literal comprehension.

In addition to testing the RH hypothesis for metaphor, an aim of the current study was to consider what difference, if any, the specific form of a metaphor makes. To address this question, we included equal numbers of nominal-entity, nominal-event, and predicate metaphor-literal sentence pairs. Intriguingly, both the group and single case statistics indicate that different types of metaphors are not equivalent. At the group level, patients found nominal-entity items the most difficult to understand and predicate items the easiest to understand, with nominal-event items intermediate in difficulty. Further, this accuracy trend was specific to the metaphoric expressions; the three types of sentences were equally easy to understand when literal. That figurative expressions involving entity nouns should be more difficult than those involving verbs presents an intriguing contrast with the difficulty of nouns and verbs when used literally. At the single word level, at least, verbs are generally more difficult to process and more fragile following injury than nouns (Damasio and Tranel, 1993; Vigliocco et al., 2011).

What might explain this contrast between the figurative and literal patterns for nouns and verbs? Our creation of metaphors of different types was originally motivated by the observation that nouns and verbs in isolation appear to draw upon different neural areas, perhaps related to the different semantic domains to which they typically refer (for review, see Cardillo et al., 2010, 2017). Nouns and verbs also play different roles in sentences. While nouns typically refer to static entities, verbs encode critical thematic role information, linking the agent, patient, and instrument of a sentence to form a coherent event. This inherent relationality renders verbs more abstract than nouns, which may empower them to be used more flexibly than nouns. Gentner and France (1988) have referred to this enhanced flexibility as the “verb mutability effect,” demonstrating that verbs more readily adjust their meanings (in literal contexts) than nouns do. Taken together, we hypothesize that verbs may more easily lend themselves to abstractions like metaphor than nouns do. Although speculative, this idea accords well with the higher frequency of predicate than nominal metaphors in corpus analyses (Pragglejaz Group, 2007).

The single case analyses also indicate metaphors of different types are processed differently. Of the five patients showing specific difficulty with metaphors, only one exhibited an impairment affecting predicate comprehension (642). The others – all left frontal cases – all showed difficulty with nominal-entity metaphors or both nominal forms, but intact predicate metaphor comprehension. Notably, the patient with impaired predicate comprehension (642) was also impaired on nominal-event metaphors. While the syntactic form differs between these two metaphor types, what they have in common is the semantic domain of their source terms (action verbs and nominalized action verbs, respectively). It is worth noting in this context that 642’s lesion encompasses posterior MTG, an area associated with action semantics in both literal and metaphoric sentences (Wallentin et al., 2005, 2011; Chen et al., 2008). The varied patterns of impairment are consistent with hypotheses that both the syntactic form and semantic domain of metaphors impacts how the metaphor network is recruited (Cardillo et al., 2010, 2017). Minimally, they indicate metaphors of different types rely on non-identical cognitive and neural mechanisms and suggest a novel area for further investigation.

More than 40 years have passed since Winner and Gardner’s (1977) seminal study of metaphor comprehension following focal brain injury, and the field is ready to move beyond questions of laterality – we contend that the right hemisphere’s putative privileged role in metaphor processing is not right. Rather, we propose that the abstraction and complex semantic manipulations required to understand metaphor may render it especially fragile in the face of injury to the left hemisphere language network.

## Author Contributions

The experiment was conceived by EC and AC. The stimuli were created by EC. The experimental tasks were administered to participants by MM. Data analysis was done by EC and MM. All authors were involved in data interpretation. The paper was written by EC. All authors approved the final version for submission.

## Conflict of Interest Statement

The authors declare that the research was conducted in the absence of any commercial or financial relationships that could be construed as a potential conflict of interest.

## Footnotes

1 We reported two of these cases (384, 642) in this initial report. However, the classification of 384 changed from one of a differential metaphor deficit to a selective metaphor deficit when comparing his behavior to the larger control group reported here.
