# Supplementary material for: Selective Metaphor Impairments After Left, Not Right, Hemisphere Injury
Source: Front Psychol. 2018 Dec 3;9:2308. doi: 10.3389/fpsyg.2018.02308 (PMC6286990; doi:10.3389/fpsyg.2018.02308)
Supplement: Supplementary file 1 [file Table_1.DOCX]

Supplementary Table1. Single Case Statistics for Patients with a Simple Metaphor Impairment

|  | | Single Bayes | | | | | | | Differential Bayes | | | | | |
| --- | --- | --- | --- | --- | --- | --- | --- | --- | --- | --- | --- | --- | --- | --- |
|  |  | Case Scores | | Significance Test | Estimated % of control population obtaining lower score than case | | Estimated effect size | | Significance Test | Estimated % of control population obtaining discrepancy more extreme than case in same direction | | Estimated effect size | | Meets criteria for Dissociation |
| Patient | Condition | Accuracy | Z-score | p | Point | 95% CI | Point | 95% CI | p | Point  (%) | 95% CI | Point | 95% CI | (Strong, Classical) |
| 360 | Literal | 42.4 | -7.300 | **0.00000** | 0.0000 | 0.0000 – 0.0000 | -7.300 | -9.6 – -5.0 | 0.0167* | 1.668 | 0.0000 – 18.363 | -3.741 | -6.765 – -0.902 | No |
|  | Metaphor | 43.1 | -5.087 | **0.00004** | 0.0043 | 0.0000 – 0.0314 | -5.087 | -6.7 –  -3.4 |  |  |  |  |  |  |
| 593 | Literal | 64.4 | -4.157 | **0.00034** | 0.0336 | 0.0000 – 0.2811 | -4.157 | -5.53 –  -2.77 | 0.0323* | 3.225 | 0.0000 – 29.038 | 2.839 | 0.552 – 5.269 | No |
|  | Metaphor | 36.2 | -5.837 | **0.0001** | 0.0009 | 0.0000 – 0.0041 | -5.837 | -7.72 –  -3.94 |  |  |  |  |  |  |
| 384 | Literal | 88.1 | -0.771 | 0.23039 | 23.039 | 10.292-39.655 | -0.771 | -1.265 – -0.262 | **0.00002** | 0.002 | 0.0000 – 0.0098 | 5.715 | 3.725 - 2147483647 | Yes, Classical |
|  | Metaphor | 51.7 | -4.152 | **0.00034** | 0.0340 | 0.0000 – 0.2842 | -4.152 | -5.526 – -2.766 |  |  |  |  |  |  |
| 493 | Literal | 94.9 | 0.200 | 0.42366 | 57.634 | 40.309 – 73.899 | 0.200 | -0.245 – 0.640 | **0.00001** | 0.001 | 0.0000 – 0.0041 | 5.776 | 3.938 - 2147483647 | Yes, Classical |
|  | Metaphor | 60.3 | -3.217 | **0.0027** | 0.2697 | 0.0008 – 1.7642 | -3.217 | -4.315 - -2.105 |  |  |  |  |  |  |
| 529 | Literal | 86.4 | -1.014 | 0.16735 | 16.735 | 6.0769-32.177 | -1.014 | -1.548 - -0.463 | **0.00073** | 0.073 | 0.0000 – 0.6358 | 4.036 | 2492 – 5.780 | Yes, Classical |
|  | Metaphor | 58.6 | -3.402 | **0.0018** | 0.1799 | 0.0003-1.2662 | -3.402 | -4.554 – -2.236 |  |  |  |  |  |  |
| 642 | Literal | 94.9 | 0.200 | 0.42366 | 57.634 | 40.309 – 73.898 | 0.200 | -0.245 – 0.640 | **0.00007** | 0.007 | 0.0000 – 0.0533 | 4.821 | 3.272 – 6.605 | Yes, Classical |
|  | Metaphor | 65.5 | -2.652 | **0.00902** | 0.9016 | 0.0166 – 4.4569 | -2.652 | -3.589 - -1.700 |  |  |  |  |  |  |
| 792 | Literal | 81.4 | -1.729 | 0.05399 | 5.3985 | 0.7777 – 15.3920 | -1.729 | -2.419 – -1.020 | 0.20411 | 20.411 | 2.6028 – 52.6022 | 0.918 | -0.065 – 1.943 | No |
|  | Metaphor | 69.0 | -2.272 | **0.012951** | 1.9510 | 0.0957 – 7.7323 | -2.272 | 03.103 - -1.423 |  |  |  |  |  |  |

*Not significant after correction for multiple comparisons
