# Supplementary material for: Selective Metaphor Impairments After Left, Not Right, Hemisphere Injury
Source: Front Psychol. 2018 Dec 3;9:2308. doi: 10.3389/fpsyg.2018.02308 (PMC6286990; doi:10.3389/fpsyg.2018.02308)
Supplement: Supplementary file 2 [file Table_2.DOCX]

Supplementary Table 2. Single Case Statistics for Different Types of Metaphor

|  | | | Single Bayes | | | | | | | Differential Bayes | | | | |
| --- | --- | --- | --- | --- | --- | --- | --- | --- | --- | --- | --- | --- | --- | --- |
|  |  |  | Case Scores | | Significance Test | Estimated % of control population obtaining lower score than case | | Estimated effect size | | Significance Test | Estimated % of control population obtaining discrepancy more extreme than case | | Estimated effect size | |
| Patient | Condition | | Accuracy | z | p | Point | 95% CI | Point | 95% CI | p | Point | 95% CI | Point | 95% CI |
| 384 | Nom-Entity | Literal | 84.2 | -.745 | 0.23800 | 23.800 | 10.843 – 40.514 | -0.745 | -1.235 – 0.240 | **0.00018** | 0.018 | 0.0000 – 0.1401 | 4.822 | 2.989 - 2147483647 |
|  |  | Metaphor | 35.0 | -5.248 | **0.00003** | 0.003 | 0.0000 – 0.0207 | -5.248 | -6.953 – 3.531 |  |  |  |  |  |
|  | Nom-Event | Literal | 85.0 | -1.131 | 0.14175 | 14.175 | 4.5777 – 28.888 | -1.131 | -1.687 – -0.557 | **0.00024** | 0.024 | 0.0000 – 0.1853 | 4.729 | 2.902 - |
|  |  | Metaphor | 36.8 | -4.560 | **0.00014** | 0.0137 | 0.0000 – 0.1138 | -4.560 | -6.057 - -3.052 |  |  |  |  |  |
|  | Predicate | Literal | 95.0 | 0.182 | 0.43052 | 56.947 | 36.646 – 73.2861 | 0.182 | -0.263 – 0.621 | 0.10463 | 10.463 | 2.6192 -23.9233 | 1.300 | 0.709 – 1.940 |
|  |  | Metaphor | 84.2 | -0.840 | 0.21114 | 21.115 | 8.9365 – 27.4487 | -0.84 | -1.345 – -0.32 |  |  |  |  |  |
| 493 | Nom-Entity | Literal | 94.7 | 0.284 | .39221 | 60.779 | 43.384 – 76.672 | 0.284 | -0.167 – 0.728 | **0.00025** | 0.025 | 0.0000 – 0.2146 | 4.334 | 2.856 -6.039 |
|  |  | Metaphor | 50.0 | -3.762 | **0.00081** | 0.081 | 0.0000 – 0.6365 | -3.762 | -5.020 – -2.491 |  |  |  |  |  |
|  | Nom-Event | Literal | 95.0 | 0.060 | 0.47714 | 52.286 | 35.2004 – 69.055 | 0.060 | -0.380 – 0.497 | **0.00005** | 0.005 | 0.0000 – 0.0324 | 5.110 | 3.411 - 2147483647 |
|  |  | Metaphor | 47.4 | -3.647 | **0.00105** | 0.1048 | 0.0001 – 0.7988 | -3.647 | -4.870 - -2.409 |  |  |  |  |  |
|  | Predicate | Literal | 95.0 | 0.182 | 0.43052 | 56.947 | 36.646 – 73.2861 | 0.182 | -0.263 – 0.621 | 0.10463 | 10.463 | 2.6192 -23.9233 | 1.300 | 0.709 – 1.940 |
|  |  | Metaphor | 84.2 | -0.84 | 0.21114 | 21.115 | 8.9365 – 27.4487 | -0.84 | -1.345 – -0.32 |  |  |  |  |  |
| 529 | Nom-Entity | Literal | 78.9 | -1.265 | .11608 | 11.608 | 3.2274 – 25.3759 | -1.265 | -1.848 – -0.663 | **0.00028** | 0.028 | 0.0000 – 0.2191 | 4.795 | 2.849 - 2147483647 |
|  |  | Metaphor | 30.0 | -5.743 | **0.00001** | 0.0011 | 0.0000 – 0.0053 | -5.743 | -7.600 – -3.875 |  |  |  |  |  |
|  | Nom-Event | Literal | 95.0 | 0.060 | 0.47714 | 52.286 | 35.2004 – 69.055 | 0.060 | -0.380 – 0.497 | 0.03163* | 3.163 | 0.2259 – 11.2073 | 1.984 | 1.216 – 2.839 |
|  |  | Metaphor | 73.7 | -1.379 | 0.09706 | 9.7061 | 2.3415 – 22.5925 | -1.379 | -1.988 - -0.752 |  |  |  |  |  |
|  | Predicate | Literal | 85.0 | -1.636 | 0.06339 | 6.339 | 1.0597 – 17.1082 | -1.636 | -2.305 – -0.950 | 0.35294 | 35.294 | 9.8917 – 67.2874 | 0.408 | -0.448 – 1.288 |
|  |  | Metaphor | 73.7 | -1.957 | 0.03566 | 3.566 | 0.3406 – 11.6765 | -1.957 | -2.706 – -1.191 |  |  |  |  |  |
| 642 | Nom-Entity | Literal | 89.5 | -0.225 | 0.41409 | 41.409 | 25.251 – 58.760 | -0.225 | -0.667 – 0.221 | 0.02499* | 2.499 | 0.0848 – 10.4078 | 2.144 | 1.259 – 3.139 |
|  |  | Metaphor | 65.5 | -2.228 | **0.02127** | 2.1273 | 0.1154 – 8.2108 | -2.228 | -3.047 – -1.391 |  |  |  |  |  |
|  | Nom-Event | Literal | 95.0 | -0.060 | 0.47714 | 52.286 | 35.2004 – 69.055 | 0.060 | -0.380 – 0.497 | **0.00906** | 0.906 | 0.0117 – 4.7372 | 2.614 | 1.671 – 3.679 |
|  |  | Metaphor | 68.4 | 0.0445 | **0.04454** | 4.4541 | 0.5329 – 13.5502 | -1.836 | -2.554 – -1.101 |  |  |  |  |  |
|  | Predicate | Literal | 100.0 | 1.091 | 0.15020 | 84.980 | 70.007 – 94.9434 | 1.091 | 0.525 – 1.639 | **0.00010** | 0.01 | 0.0000 – 0.0783 | 4.595 | 3.162 -6.250 |
|  |  | Metaphor | 68.4 | -2.521 | 0.01181 | 1.1811 | 0.0312 – 5.4224 | -2.521 | -3.421 – -1.605 |  |  |  |  |  |
| 792 | Nom-Entity | Literal | 68.4 | -2.294 | **0.01867** | 1.8665 | 0.0869 – 7.4977 | -2.294 | -3.132 – -1.440 | 0.18587 | 18.587 | 1.0550 – 55.8068 | 1.042 | -0.146 – 2.306 |
|  |  | Metaphor | 55.0 | -3.267 | **0.00242** | 0.2418 | 0.0006 – 1.6152 | -3.267 | -4.380 – -2.141 |  |  |  |  |  |
|  | Nom-Event | Literal | 80.0 | -1.726 | 0.05621 | 5.421 | 0.7841 – 15.4346 | -1.726 | -2.416 – -1.018 | 0.44430 | 44.43 | 15.4177 – 76.0251 | 0.152 | -0.707 – 1.109 |
|  |  | Metaphor | 68.4 | -1.836 | **0.04454** | 4.454 | 0.5329 – 13.5502 | -1.836 | -2.554 – -1.101 |  |  |  |  |  |
|  | Predicate | Literal | 95.0 | 0.182 | 0.42052 | 56.948 | 39.646 – 73.2861 | 0.182 | -0.263 – 0.621 | 0.10463 | 10.463 | 2.6192 – 23.9233 | 1.300 | 0.709 – 1.940 |
|  |  | Metaphor | 84.2 | -0.840 | 0.21114 | 21.115 | 8.9365 – 37.448 | -0.84 | -1.345 – -0.320 |  |  |  |  |  |

*Not significant after correction for multiple comparisons
